# New-onset atrial fibrillation in sepsis is associated with increased morbidity and mortality

**DOI:** 10.1007/s12471-014-0641-x

**Published:** 2015-01-09

**Authors:** Sumeet Gandhi, Dhanjit Litt, Neeraj Narula

**Affiliations:** 1 McMaster University, Division of Cardiology, Hamilton, Ontario Canada Hamilton Health Sciences Centre, 237 Barton Street East, Office 329, 3 Lower North, L8L 2X2 Hamilton, Ontario Canada; 2 University of Toronto, Division of Internal Medicine, Toronto, Ontario, Canada Toronto General Hospital, 200 Elizabeth Street, M5G 2C4 Toronto, Ontario Canada; 3 McMaster University, Division of Gastroenterology, Hamilton, Ontario, Canada Hamilton Health Sciences Centre, 237 Barton Street East Office 329, 3 Lower North, Hamilton, Ontario Canada

**Keywords:** Atrial fibrillation, Arrhythmia, Sepsis, Intensive care unit, Mortality

## Abstract

**Background:**

The development of new-onset atrial fibrillation in sepsis has been associated with adverse outcomes.

**Methods:**

A systematic literature search was conducted to retrieve articles that investigated the association of new-onset atrial fibrillation in patients diagnosed with sepsis. The primary outcome of interest was the pooled risk ratio (RR) of in-hospital mortality in patients with new-onset atrial fibrillation and sepsis.

**Results:**

Six studies included 3100 patients with new-onset atrial fibrillation in sepsis and 36,900 patients without new-onset atrial fibrillation in sepsis. The pooled RR for in-hospital mortality was 1.45 (95 % CI 1.32–1.60, *p* < 0.00001, *I* ^2 = ^24 %). New-onset atrial fibrillation was also associated with increased ICU mortality, ICU and in-hospital length of stay and stroke. New-onset atrial fibrillation occurred more in the elderly, those with a prior history of cardiovascular and respiratory disease, and those with increased severity of illness.

**Conclusion:**

Prospective randomised trials are needed to clarify the significance of new-onset atrial fibrillation in sepsis, optimal treatment strategies for these patients, and the benefit of systemic anticoagulation. Physicians should be aware that new-onset atrial fibrillation in sepsis is not merely an observed temporary arrhythmia but a marker of poor prognosis and should be managed accordingly.

## Introduction

Sepsis represents a significant proportion of morbidity and mortality in the intensive care unit (ICU). Despite advancements in therapy, mortality rates far exceed those of other medical conditions such as myocardial infarction and stroke. The development of new-onset atrial fibrillation is an indicator of poor prognosis in patients with critical illness. Atrial fibrillation occurs in up to 40 % of patients post coronary artery bypass graft implantation and up to 50 % post cardiac valve surgery [1, 2]. Prospective registries have demonstrated that postoperative atrial fibrillation is associated with a threefold increase in postoperative complications such as myocardial infarction, heart failure, acute respiratory failure, and stroke.

## Mechanism of new-onset atrial fibrillation in sepsis

The proposed mechanism for atrial fibrillation in sepsis involves the production of an anatomical and electrophysiological substrate as a consequence of a hyper-inflammatory state [3−5]. Multiple imaging modalities demonstrate that patients with sepsis experience biventricular dilatation with both systolic and diastolic dysfunction. These changes are unrelated to coronary perfusion, and it is suggested that circulating cytokines and local production of cardio-depressant factors are the underlying cause of this *septic cardiomyopathy* [6−8]. Using an in-vitro myocardial assay, the effects of inflammatory cytokines derived from the serum of humans with septic shock were examined to assess its effects on myocardial contractile function. Independently and synergistically, tumour necrosis factor-alpha and interleukin-1b showed a concentration-dependent depression in myocardial contractility; removal of both cytokines resulted in the elimination of serum myocardial depressant activity.

In the setting of this hyper-inflammatory state, the combination of depressed myocardial function and large volume fluid resuscitation may result in an acute increase in left ventricular end-diastolic pressure and subsequent left atrial stretch, in turn providing an anatomical substrate upon which atrial fibrillation can occur. Furthermore, an independent process of ventricular remodelling due to sepsis may decrease ventricular chamber compliance and may further alter left atrial and pulmonary venous haemodynamics. Support for this theory stems from population-based studies that confirm the importance of an anatomical substrate in the generation of atrial tachyarrhythmia [9].

In addition, there is evidence to suggest that systemic inflammation in sepsis induces an electrophysiological substrate for atrial fibrillation. Aoki et al. investigated the role of ion channels in sepsis-induced atrial tachyarrhythmia. Sepsis was induced in guinea pigs through inoculation of lipopolysaccharide (LPS), an endotoxin from the cell wall of gram-negative organisms and known potent inducer of the systemic inflammatory cascade [10]. Post inoculation, atrial cells isolated from LPS-treated animals demonstrated significantly shortened action potential duration. These changes were associated with reduced L-type calcium current and an increased delayed rectifier potassium current. Inducible nitric oxide synthase was found to be upregulated, and atrial nitric oxide production was increased. The changes in action potential duration were reversed when LPS was co-administered with inhibitors of nitric oxide synthase. A shortened action potential duration in the setting of sepsis may reflect an inflammation-induced nitration of ion channels which may contribute to the development of sepsis-induced atrial fibrillation. Future studies are needed to explore these proposed mechanisms.

Recent studies have investigated the association of new-onset atrial fibrillation in patients with sepsis. New-onset atrial fibrillation has been associated with longer stay in hospital, and overall increased mortality. We provide a systematic review and meta-analysis of studies describing increased morbidity and mortality in patients with new-onset atrial fibrillation and sepsis.

## Methods

### Study selection

A systematic search was conducted to retrieve articles that investigated the association of new-onset atrial fibrillation in patients diagnosed with sepsis. We identified potential English-language sources from the PubMed, Medline, and EMBASE databases from the year 1950 to December 2013. Keywords used were “atrial fibrillation” and (“sepsis” or “septic shock”). In addition, reference lists of any studies meeting inclusion criteria were reviewed manually to identify additional relevant publications.

### Inclusion criteria

Studies were included that met the following criteria: (i) observational studies that evaluated patients with new-onset atrial fibrillation with a diagnosis of sepsis or septic shock; (ii) patients who were admitted to a medical or surgical intensive care unit; (iii) studies that include a control group of patients with a diagnosis of sepsis without new-onset atrial fibrillation; (iv) studies that are published as a full article in the English language. Eligibility assessment and data extraction were carried out independently by two investigators (SG and DL) with discrepancies resolved by consensus in consultation with the senior author.

### Outcomes of interest

The primary outcome of our meta-analysis was the pooled relative risk ratio (RR) of in-hospital mortality with a diagnosis of new-onset atrial fibrillation in sepsis. Meta-analysis was conducted by combining the risk ratios of individual studies into a pooled RR using a random-effects model. Relative RRs are reported with 95 % confidence intervals (CIs). We tested for heterogeneity using the chi-squared test and the *I* ^2^ test. The *I* ^2^ test describes the percentage of variability in effect estimates that is due to heterogeneity rather than chance. A value of 25 % suggests low variability, 50 % suggests moderate variability, and 75 % suggests high variability between studies [11]. Sensitivity analysis was conducted for outcomes reported, funnel plots were constructed to assess for publication bias. Analyses was performed with RevMan 5.1 (Review Manager (RevMan) [Computer program]. Version 5.1. Copenhagen: The Nordic Cochrane Centre, The Cochrane Collaboration, 2011).

### Study quality and data extraction

Quality assessment was carried out independently by two investigators (SG and DL) using the Newcastle-Ottawa quality assessment scale. Our assessment included studies based on three aspects: the selection of the study groups (0–4 points), the comparability of the groups (0–2 points), and the ascertainment of either the exposure or outcome of interest (0–3 points), with a maximum total score of 9. A score ≥ 5 indicated adequate quality for inclusion in the systematic review**.** Discrepancies in interpretation of data and inclusion of studies were resolved by consultation with the senior author.

## Results

### Search results

**Fig. 1:**
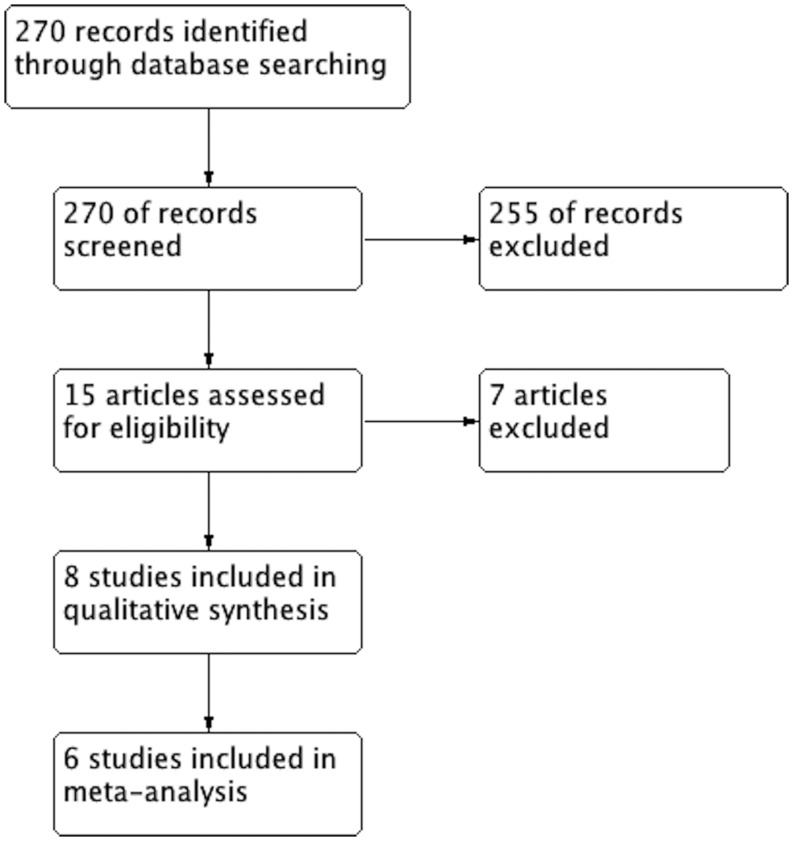
Study flow diagram

Our search strategy yielded 270 studies, of which 255 were excluded on review of the title and abstract (Fig. 1). A further seven studies were excluded after careful review of the full text. Four narrative review articles were excluded as no new patient data were offered [12−15]. Seguin et al. performed a prospective observational study of trauma patients who were admitted to a surgical ICU [16]. New-onset atrial fibrillation was associated with a diagnosis of sepsis, and demonstrated increased ICU and hospital length of stay (*p* < 0.001). This study was excluded, as it could not provide specific outcomes for patients diagnosed with new-onset atrial fibrillation and sepsis. Arora et al. performed a single-centre, prospective observational study of patients admitted to a medical-surgical ICU [17]. Admission for sepsis was an independent risk factor for the development of new-onset atrial fibrillation (OR 4.87, *p* = 0.02) and patients in the new-onset atrial fibrillation group had higher in-hospital mortality (RR 2.7, *p* < 0.05). This study was excluded, as the authors could not provide specific outcomes for patients diagnosed with sepsis and new-onset atrial fibrillation. Kindem et al. retrospectively evaluated patients admitted with bacteraemia, and found that new-onset atrial fibrillation was associated with increased 14-day mortality. This study was excluded as only 16 % of patients with new-onset atrial fibrillation met the systemic inflammatory response syndrome (SIRS) criteria, therefore not meeting the criteria for sepsis [18].

Salman et al. [19] assessed outcomes in patients with new-onset atrial fibrillation admitted to a surgical ICU. The authors of this study were contacted and provided the information specifically for patients with a diagnosis of sepsis and the development of new-onset atrial fibrillation.

Overall eight studies were eligible for inclusion in the systematic review [19−26]. Table 1 summarises characteristics for each individual study, Table 2 summarises patient characteristics, and Table 3 summarises study outcomes. We assessed the quality of included studies using the Newcastle-Ottawa quality assessment scales**.** Overall, the quality of studies was quite high. Two studies scored nine points [20, 23], one study scored eight points [19], one study scored seven points [21], three studies scored six points [24−26], and one study scored five points [22].

**Table 1:** Study characteristics

Author (year)	Study design	Inclusion	Exclusion	Quality of study^a^
Walkey et al. [25]	Retrospective	California State Independent database claims, adults hospitalised with severe sepsis	*n/a*	6
Wells et al. [26]	Retrospective	Admitted to the medical ICU with sepsis	Recent myocardial injury, malignancy	6
Kumar et al. [22]	Retrospective	Nationwide Inpatient Sample 2007 database, adults diagnosed with severe sepsis or septic shock	*n/a*	5
Meierhenrich et al. [23]	Prospective	Admitted to general surgical ICU, diagnosed with septic shock	If underwent cardiac surgery, previous history of known AF	9
Goodman et al. [12]	Prospective	Admitted to general surgical ICU, diagnosed with sepsis	Recent thoracic surgery, sustained thoracic trauma	7
Salman et al. [19]	Retrospective	Admitted to a medical-surgical ICU diagnosed with sepsis, severe sepsis, septic shock	Previous AF	8
Christian et al. [20]	Retrospective	Admitted to a medical-surgical ICU diagnosed with sepsis	If underwent cardiothoracic injury, acute MI, acute pulmonary embolism	9
Seguin et al. [24]	Prospective	Admitted to a surgical ICU	AF on admission, permanent pacemaker	6

^a^Newcastle-Ottawa assessment scale. n/a not applicable, *AF* atrial fibrillation, *ICU* intensive care unit, *MI* myocardial infarction

**Table 2:** Patients characteristics

Author (year)	Mean age (years), female		Comorbidities	
	New-onset AF	Control	New-onset AF	Control
Walkey et al. [25]	74, 44 %	66, 49 %	Hypertension: 46 %	Hypertension: 48 %
			Diabetes mellitus: 28 %	Diabetes mellitus: 34 %
			CAD: 6 %	CAD: 5 %
			Stroke: 4 %	Stroke: 2 %
			CHF: 11 %	CHF: 6 %
			COPD: 7 %	COPD: 5 %
Wells et al. [26]	72, 41 %	62, 45 %	CAD: 40 %	CAD: 19 %
			COPD: 39 %	COPD: 29 %
			Diabetes Mellitus: 41 %	Diabetes Mellitus: 33 %
Kumar et al. [22]	n/a	n/a	n/a	n/a
Meierhenrich et al. [23]	66, 22 %	56, 44 %	Hypertension: 74 %	Hypertension: 41 %
			CAD: 22 %	CAD: 7 %
			CHF: 4 %	CHF: 0 %
			COPD: 17 %	COPD: 4 %
Goodman et al. [12]	69, 50 %	53, 42 %	Hypertension: 35 %	Hypertension: 14 %
			Diabetes mellitus: 37 %	Diabetes mellitus: 7 %
			CAD: 31 %	CAD: 35 %
			CHF: 4 %	CHF: 15 %
			COPD: 7 %	COPD: 23 %
Salman et al. [19]	67, 28 %	56, 50 %	Hypertension: 44 %	Hypertension: 38 %
			Diabetes mellitus: 16 %	Diabetes mellitus: 27 %
			CAD: 8 %	CAD: 4 %
			Stroke: 8 %	Stroke: 4 %
			COPD: 4 %	COPD: 2 %
Christian et al. [20]	66	62.2	n/a	n/a
Seguin et al. [24]	57, 13 %	41, 24 %	Hypertension: 6 %	Hypertension: 10 %
			CAD: 6 %	CAD: 3 %
			Chronic lung disease: 6 %	Chronic lung disease: 4 %

*CAD* coronary artery disease, *COPD* chronic obstructive pulmonary disease, *CHF*congestive heart failure

**Table 3:** Study outcomes

Author (year)	Significant results		
Walkey et al. [25]	New-onset AF in sepsis vs. sepsis alone	In-hospital ischaemic stroke	OR 2.70 (2.05–3.57, *p* < 0.001)
		In-hospital mortality	RR 1.07 (1.04–1.11, *p* < 0.001)
Wells et al. [26]	New-onset AF in sepsis vs. sepsis alone	In-hospital mortality	72 % vs 57 %, *p* < 0.0001
Kumar et al. [22]	New-onset AF In-hospital mortality of new-onset AF and severe sepsis or septic shock vs. severe sepsis or septic shock alone	Frequency in severe sepsis	OR 1.1 (1.13–1.19, *p* < 0.05)
		Frequency in septic shock	OR 1.33 (1.29–1.37, *p* < 0.05)
		Severe sepsis	OR 1.19 (1.14–1.24, *p* < 0.05)
		Septic shock	OR 1.12 (1.07–1.18, *p* < 0.05)
Meierhenrich et al. [23]	New-onset AF in severe sepsis vs. sepsis alone	ICU mortality	39 % vs. 22 %, *p* = 0.14
		28-day mortality	39 % vs. 22 %, *p* = 0.22
		60-day mortality	48 % vs. 26 %, *p* = 0.14
		Median ICU LOS (days)	30 vs. 17, *p* = 0.017
Goodman et al. [12]	New-onset SVA and sepsis vs. sepsis alone	In-hospital mortality	OR 1.99 (1.09–3.64, *p* = 0.026)
Salman et al. [19]	Paroxysmal AF and sepsis vs. sepsis alone	ICU mortality	48 % vs. 27 %, *p* = 0.0614
		In-hospital mortality	64 % vs. 36 %, *p* = 0.018
		28-day mortality	72 % vs. 38 %, *p* = 0.041
		Median ICU LOS (days)	OR 3.284 (1.126–9.574, *p* = 0.0294) 8 vs. 3, *p* = 0.0087
Christian et al. [20]	New-onset AF and sepsis vs. sepsis alone	ICU mortality	68.8 % vs. 39.8 %, *p* = 0.034
		Median ICU LOS (days)	27.8 vs. 7.6, *p* = 0.0001
		Hospital LOS (days)	63.4 vs. 29.4, *p* = 0.047
		Mechanical ventilation (days)	23.58 vs. 8.35, *p* = 0.006
Seguin et al. [24]	New-onset AF and sepsis vs. sepsis alone	In-hospital mortality	25 % vs. 11 %

*AF*  atrial fibrillation,  *ICU*  intensive care unit, *LOS*   length of stay, *OR*  odds ratio, *RR*   relative risk*, SVA*  supraventricular arrhythmia

### Meta-analysis of in-hospital mortality

**Fig. 2:**
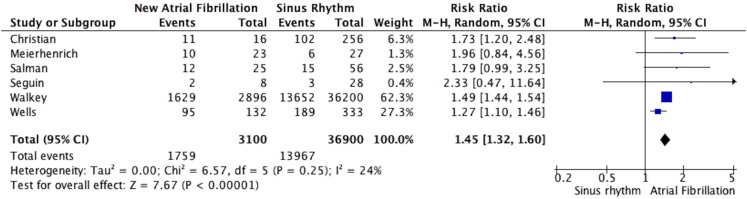
In-hospital mortality of patients with new-onset atrial fibrillation and sepsis

Six studies met inclusion criteria for assessing in-hospital mortality in new-onset atrial fibrillation in patients with sepsis. Fig. 2 summarises the results of studies of in-hospital mortality in new-onset atrial fibrillation and sepsis. A total of 3100 patients with new-onset atrial fibrillation in sepsis were included, and 36,900 patients without new-onset atrial fibrillation in sepsis. A total of 1759 (57 %) patients with new-onset atrial fibrillation and sepsis experienced in-hospital mortality, compared with 13,967 (38 %) without new-onset atrial fibrillation and sepsis. The pooled RR for in-hospital mortality in patients with new-onset atrial fibrillation was 1.45 (95 % CI 1.32–1.60, *p* < 0.00001, *I* ^2 = ^24 %), demonstrating a significantly increased in-hospital mortality. Sensitivity analysis showed the results remained after removal of each study.

### Morbidity and mortality

Eight studies met the inclusion criteria: five retrospective studies and three prospective studies. Walkey et al. performed a retrospective study of hospitalised patients with severe sepsis. Patients with new-onset atrial fibrillation in sepsis compared with sepsis with sinus rhythm were older (mean age of 74 years compared with 66 years) and had increased risk of in-hospital mortality with a combined multivariate adjusted RR 1.07 (95 % CI 1.04–1.11, *p* < 0.001) [25]. A single-centre, retrospective study by Wells found patients with new-onset atrial fibrillation in sepsis compared with sinus rhythm were older (mean age 72 years compared with 62 years, *p* < 0.0001), and were more likely to have coronary artery disease (*p* < 0.0001) [26]. In-hospital mortality was significantly increased in patients with new-onset atrial fibrillation and sepsis compared with sepsis alone (72 % vs. 57 %, *p* < 0.0001). Kumar et al. published results of their retrospective, observational study and found that new-onset atrial fibrillation compared with sinus rhythm was associated with increased mortality in patients with severe sepsis (OR 1.19, 95 % CI 1.14–1.24, *p* < 0.05) and septic shock (OR 1.12, 95 % CI 1.07–1.18, *p* < 0.05) [22]. Meierhenrich performed a single-centre, prospective observational study of patients admitted to a general surgical ICU with a diagnosis of septic shock excluding those who underwent cardiac surgery [23]. Patients with new-onset atrial fibrillation compared with sinus rhythm were older (mean age of 66 years compared with a mean age of 56 years, *p* < 0.01) and frequently had a history of hypertension (*p* = 0.02). There was no difference between doses and frequency of vasopressors, SAPS II score, and serum electrolytes between both groups. Mortality was increased in patients with new-onset atrial fibrillation and septic shock compared with sepsis with sinus rhythm, however did not reach statistical significance. Salman et al. performed a single-centre, retrospective cohort study of patients admitted to a medical-surgical ICU with a diagnosis of sepsis [19]. Patients with new-onset atrial fibrillation had higher APACHE III scores (*p* = 0.0091) and lower mean ejection fractions (48.9 % vs. 59.1 %, *p* = 0.0322). The atrial fibrillation group demonstrated increased in-hospital mortality (64 % vs. 36 %, *p* = 0.018), 28-day mortality (72 % vs. 38 %, *p* = 0.041), and median length of ICU stay (8 days vs. 3 days, *p* = 0.0087). Multiple logistic regression analysis demonstrated that new atrial fibrillation was associated with increased risk of mortality at 28 days (OR 3.284, 95 % CI 1.126–9.574, *p* = 0.0294). Christian et al. performed a single-centre, retrospective observational study of patients admitted to a medical-surgical ICU with a diagnosis of sepsis [20]. Patients with sepsis and new-onset atrial fibrillation compared with sinus rhythm had increased ICU mortality (68.8 % vs. 39.8 % *p* = 0.034). Patients with new-onset atrial fibrillation and sepsis had increased ICU length of stay (27.8 days vs. 7.6 days, *p* = 0.0001), increased hospital length of stay (63.4 days vs. 29.4 days, *p* = 0.047), and increased need for mechanical ventilation (23.58 ventilation days vs. 8.35 ventilation days. *p* = 0.006). Goodman et al. performed a prospective, single-centre study of patients admitted to a surgical ICU excluding those who underwent recent thoracic surgery or sustained thoracic trauma [21]. New-onset supraventricular arrhythmia (SVA) in patients with sepsis was associated with an increased risk of in-hospital mortality (OR 1.99, 95 % CI 1.09–3.64, *p* = 0.026) and significantly longer hospital stays (*p* < 0.01) independent of age. After four years of follow-up, overall mortality was higher in patients with new-onset SVA; however, most of these deaths occurred in the first year post hospitalisation. Seguin et al. performed a single-centre, prospective observational study of patients admitted to a surgical ICU excluding those with prior documented atrial fibrillation or a permanent pacemaker [24]. New-onset atrial fibrillation was associated with increased age (*p* = 0.001), history of cardiovascular disease (*p* = 0.02), and use of calcium channel blockers (*p* = 0.002). Of patients with new-onset atrial fibrillation and sepsis, 25 % died in hospital compared with 11 % of patients with sepsis and sinus rhythm.

### Stroke

In the study by Walkey et al. [25], 75 of 2896 (2.6 %) patients with new-onset atrial fibrillation and sepsis suffered from in-hospital ischaemic stroke (multivariable adjusted OR 2.70, 95 % CI 2.05–3.57, *p* < 0.001). Multivariate analysis showed patients with new-onset atrial fibrillation had a greater risk of stroke than those with pre-existing atrial fibrillation (OR 3.63, 95 % CI 2.51–5.25, *p* < 0.001). New-onset atrial fibrillation in sepsis compared with sepsis with sinus rhythm demonstrated a trend towards increased risk of re-hospitalisation with incident ischaemic stroke (multivariable analysis HR 1.51, 95 % CI 0.98–2.33, *p* = 0.06).

### Rhythm versus rate control

Meierhenrich et al. treated 49 of 50 patients with new-onset atrial fibrillation in sepsis [23]. Electrical cardioversion was performed with adjuvant pharmacological agents in 17 of 49 patients. Amiodarone was used most frequently in 36 patients, and beta-blockers in 25 patients indicating that the majority of patients received a combination of antiarrhythmic drugs. In 42 of the 49 patients with new-onset atrial fibrillation, sinus rhythm was successfully reconstituted. Failure to restore sinus rhythm was associated with increased ICU mortality, as 5 of the 7 patients who could not maintain sinus rhythm died compared with 9 of the 42 patients who were successfully converted to sinus rhythm (*p* = 0.015).

## Discussion

The studies presented demonstrate that new-onset atrial fibrillation is associated with increased ICU mortality, in-hospital mortality, ICU length of stay, in-hospital length of stay, stroke, and adverse outcomes post discharge. New-onset atrial fibrillation occurred more in elderly patients, those with a prior history of cardiovascular and respiratory disease, and those with increased severity of illness. The study by Meierhenrich et al. suggests that in the ICU setting, rhythm control may be desirable given the increased mortality rate among those unable to achieve sinus rhythm.

Our study is the first systematic review in the literature to describe the association of new-onset atrial fibrillation with poor outcomes in patients with sepsis. This includes clinical characteristics and risk factors for adverse outcomes, as well as insight into the development of in-hospital and long-term stroke, and possible benefit of rhythm control. The strength of this study is the pooled analysis of 3100 patients that demonstrates increased in-hospital mortality in this patient population.

There are several limitations to the interpretation of our systematic review and meta-analysis. The retrospective observational nature of these studies limits our ability to assess for covariates of increased mortality. Due to the design of these studies, it is unclear what the primary diagnosis or preceding illness was before being labelled as ‘sepsis’, the source of the infection or responsible organisms, specific details around mortality, as well as the management strategy. In our pooled analysis of in-hospital mortality, Walkey et al. included 39,096 subjects which comprised 94 % of the subjects; however, the results remained after sensitivity analysis. This confirms that our results are representative to the population of the subjects included. In regards to an association of increased stroke, there are several limitations to the interpretation of this analysis due to data extraction based upon billing codes, as well as the lack of insight regarding anticoagulation practices during the admission and upon discharge of the patients.

Prospective randomised trials are needed to help clarify the significance of the development of atrial fibrillation in sepsis, and to assess the optimal treatment strategies for patients with atrial fibrillation in the ICU. Current guidelines do not offer recommendations regarding anticoagulation for stroke risk reduction in patients with new-onset atrial fibrillation in sepsis. Future datasets need to be analysed to fully assess the risk of stroke in this patient population and to assess the benefit of systemic anticoagulation alongside the risk of adverse bleeding. New-onset atrial fibrillation in septic patients is likely a marker of poor prognosis and increased mortality. Measures to improve outcomes such as maintaining adequate perfusion pressure with sinus rhythm and reducing the risk of stroke with anticoagulation should be considered in the management of patients with new-onset atrial fibrillation in the ICU. Physicians should be aware that new-onset atrial fibrillation in sepsis is not merely an observed temporary arrhythmia but is a marker of poor prognosis and should be managed accordingly.

### Disclosures

The authors do not believe that they have any conflicts of interest with regards to this research paper. This article represents original work and is not under consideration for publication elsewhere. All authors meet criteria for authorship.
